# Rapid Detection of Infectious Flacherie Virus of the Silkworm, *Bombyx mori*, using RT-PCR and Nested PCR

**DOI:** 10.1673/031.013.12001

**Published:** 2013-11-05

**Authors:** Shyam Kumar Vootla, Xing Meng Lu, Neetha Kari, Mallikarjun Gadwala, Qineng Lu

**Affiliations:** 1 Post Graduate Department of Biotechnology and Microbiology, Karnatak University, Dharwad, India; 2 Post Graduate Department of Sericulture, Karnatak University, Dharwad, India; 3 Lab of Invertebrate pathology, College of Animal Science, Zhejiang University, Hangzhou, China

**Keywords:** BmIFV, diagnosis

## Abstract

In this study, a method for detection of an ssRNA viral pathogen that causes viral flacherie in the silkworm, *Bombyx mori* (L.) (Lepidoptera: Bombycidae), was used for the detection of *B. mori* infectious flacherie virus (BmIFV). A combination of nested and reverse transcriptase polymerase chain reaction was used for detection. Although BmIFV has been reported in almost all the sericultural regions of the world, there had been no reports of BmIFV incidence in India. Therefore, the confirmation of the presence of BmIFV in Karnataka, India, is of great significance. The present method is advantageous because it can be used to detect the virus by using samples from infected midgut tissues, thus simplifying and avoiding laborious genome isolation procedures. This method could help in early detection of BmIFV disease pathogens and help reduce crop losses.

## Introduction

Silk production is an important industry in India, making the silkworm, *Bombyx mori* (L.) (Lepidoptera: Bombycidae), economically important (Bongale et al. 1991). Karnataka is the one of the leading silk producers in India, and much of the sericulture activity in Karnataka is restricted to southern most part. After the implementation of sericulture extension programs aided by World Bank funds, sericulture has moved to nontraditional belts, such as the northern districts of Karnataka, which includes the districts of Raichur, Gulbarga, Dharwad, Belgaum, Bidar etc. These regions are identified by their hot climates and high rates of occurrence of bacterial and other diseases. Silkworms are susceptible to a variety of diseases, and viral diseases are no exception (Samson et al. 1990; Priyadharshini et al. 2008). In managing viral diseases, it is difficult to identify the pathogens causing the diseases and symptoms at early stages of the disease. *B. mori* Infectious flacherie virus (BmIFV) is a single stranded RNA virus that causes flacherie-like symptoms, is the most frequent virus in *B. mori*, and is known to cause crop losses of up to 40% (Savanurmath et al. 1994). Several other kinds of viruses thought to be BmIFV have also been reported from Karnataka (Patil et al. 1982; Hadimani et al. 1983). The synergism between both bacterial and viral diseases that magnify crop losses due to viral infection is poorly understood (Ayuzawa et al. 1968). Even though BmIFV is claimed to be prevalent in India (Sanakal et al. 1996), there have been no positive confirmations of the disease. Our most recent studies are based on the existing literature and rely on the accessibility of Gene bank resources from Japan and China for the completely sequenced genomes of BmIFV.

The conventional method of BmIFV detection is the pyronine staining method, which detects A and B type bodies in histological observation, but this method is time consuming and is not a confirmative test. The preferred technique is polymerase chain reaction (PCR). The applications of PCR are various, manifold, and well-documented (Lo 1998). While the PCR technique is basically a primer extension reaction used for amplifying specific nucleic acids *in vitro*, reverse transcriptase (RT) PCR is a sensitive technique used for mRNA detection, is semi-quantitative, and is employed for converting mRNA to complementary DNA (cDNA) via reverse transcriptase (Mallet et al. 1995). It is more advantageous than northern blot analysis and RNase protection techniques as it can quantify mRNA levels from much smaller samples. This technique is sensitive enough to enable quantization of RNA from a single cell (Croci et al. 1999; Li et al. 2010).

The nucleic acid of BmIFV is an ssRNA virus (Kawase et al. 1980), and since RNA cannot serve as a template for PCR, reverse transcription combined with PCR is used to convert RNA into its complementary DNA, which is suitable for PCR. The combination of both techniques is colloquially referred to as RTPCR. The process of RT-PCR has proved valuable for detecting gene expressions, amplifying DNA sequences prior to sub cloning, and analysis and diagnosis of infectious agents or genetic diseases (Paz Sánchez-Seco et al. 2001).

## Materials and Methods

TaKaRa One-Step RNA PCR Kit (AMV) DRR024A was purchased from Takara Bio (www.takara-bio.com), and Trizol was purchased from Invitrogen (www.invitrogen.com). Isopropyl alchohol and other routine chemicals used for agarose gel electrophoresis were procured from local manufacturers.

### Virus source

Diseased *B. mori* larvae with flacherie symptoms were collected from sericulture farmers in northern districts of Karnataka, India. Furthermore, larvae infected with bacteria (with putrified debris), nuclear polyhedrosis virus (with milky hemolymph), and cytoplasmic polyhedrosis virus (with rectal protrusion) were discarded, and the ones with symptoms of viral flacherie were retained. The midguts of the diseased larvae were homogenized to collect the crude extract, and this extract was used for further propagation of the virus.

### Multiplication or propagation of the virus

*B. mori* were reared on fresh mulberry leaves in the laboratory at optimum conditions of 26° C and 65% RH. The gut extract of the diseased *B. mori* was incubated at pH 4 for 15 min at room temperature, and then neutralized with 1 mol/L NaOH, and fed to *B. mori* larvae as leaves smeared with the extract. The densonucleosis virus (DNV)-resistant *B. mori* race Feng1X54A was used for the propagation of the virus. The larvae were infected during the third instar and reared until the fifth instar, depending on the virulence of the disease; symptoms were usually seen after 4–6 days. Maximum mortality of the larvae was noticed on the 10^th^ day post infection. Multiplication of the virus was performed for several generations until uniform symptoms were seen in the *B. mori* larvae infected with the virus. *B. mori* with typical flacherie symptoms were dissected to collect the midguts, which were the site of viral infection and used for further extraction of the virus. The collected infected midguts were stored in a refrigerator at -20° C until further use.

### Purification of the virus

The virus from flacherie-infected *B. mori* larvae were isolated and purified by using the procedures of Himeno et al. (1974) and Nakagaki et al. (1987) as follows. The infected midgut tissue was homogenized in 0.05 mol/L Tris-HCl buffer (pH 7.2), and then the homogenate was centrifuged at 3000 g for 10 min. Then, the supernatant was mixed with an equal volume of chloroform and shaken vigorously. Next, the solution was centrifuged at 3000 g for 10 min to collect supernatant, and then the supernatant was saturated to 40 % with solid (NH_4_)_2_SO_4_. Next, the solution centrifuged at 12000 g for 30 min and was suspended in 0.1 mol/L EDTA Tris-HCl buffer (pH 7.2). Then, the supernatant was ultracentrifuged at 130000 g for 2 hr. The sediment thus formed was the crude virus.

### Extraction of RNA from the virus

The glassware, equipment, and buffers used for the experiment were immersed in 0.1% DEPC for 12 hr at 37° C and then autoclaved. For extracting RNA, 50 µL of virus was mixed with 1 mL Trizol reagent, and the mixture was then gently shaken and kept at 26° C for 15 min. 200 µL of chloroform was added to the mixture, which was then shaken and incubated at 28° C for 3 min. The virus suspension was then centrifuged at 12000 rpm for 15 min at 4° C. Following centrifugation, the mixture separated into phases, with a colorless aqueous layer containing the RNA. The fluid containing RNA was transferred into Eppendorf tubes. 500 µL of isopropyl alcohol was added to precipitate the RNA, and the samples were then incubated at 26° C for 15 min. This was followed by another centrifugation at 12000 rpm for 10 min at 4° C. Then, the supernatant was removed, and the RNA was washed once with 1 mL of 75% alcohol followed by centrifuging at 7500 rpm for 5 min at 4° C. The supernatant was decanted and the pellet was dried. Finally, the pellet was redissolved in 40 µL of RNAse free water. The concentration of the extracted RNA was measured using an Ultrospec 3100 Pro UV/Visible spectrophotometer (GE Healthcare Life Sciences, www.gelifesciences.com) and was used for further RT-PCR experiments.

**Table 1.**
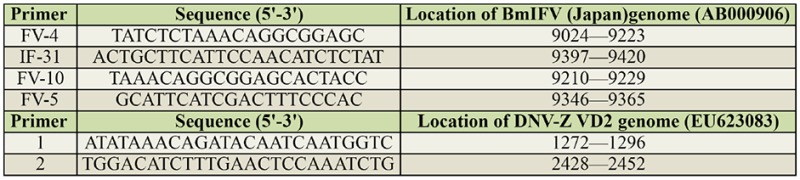
Primers for nested RT-PCR.

### Primer designing

Primers for the nested RT-PCR assay were designed based on the RNA dependent RNA polymerase and 3 untranslated regions (Table 1) of fully sequenced BmIFV (Isawa et al. 1998; Li et al. 2010). Multiple alignments were carried out to identify conserved regions using ClustalW (Tamura et al. 2007). Primers were designed manually from the alignment file.

### Positive control RNA and one-step RTPCR

The positive control RNA RT-PCR using TaKaRa One-Step RNA PCR Kit (AMV) was performed following the manufacturers protocol. Each reaction mixture was prepared adding the following components in PCR tubes: 10× one-step RNA PCR buffer (5µL), 25 mM MgCl2 (10 µL), 10 mM dNTP Mixture (5 µL), Rnase inhibitor (1 µL at 40 U/µL), AMV Rtase XL (1 µL at 5 U/µL), AMVOptimized Taq (1 µL at 5 U/µL), 20 µM Control F-1 Primer (1 µL), 20 µM Control R-1 Primer (1 µL), Positive Control RNA (1 µL), Rnase Free dH_2_O (24 µL). The control primers used were Control F-1 Primer 5´ - CTGCTCGCTTCGCTACTTGGA-3´ and Control R-1 primer 5´ - CGGCACCTGTCCTACGAGTTG-3´

PCR was performed for 30 cycles, with RT reaction at 50° C for 15 min, RTase inactivation at 94° C for 2 min, and followed by denaturation at 94° C for 30 sec, annealing at 60° C for 30 sec, and extension at 72° C for 1.5 min.

### Nested RT-PCR using virus RNA

RT-PCR was performed by using RNA from purified BmIFV and BmIFV-infected tissue, and DNA from purified DNV virus and DNV infected tissue. TaKaRa One-Step RNA PCR Kit (AMV) was used following the manufacturers protocol as described earlier. Virus RNA (≤ 1 µg total RNA) with the following primers was used for the first PCR: IF-31 ACTGCTTCATTCCAACATCTCTAT and FV-4 TATCTCTAAACAGGCGGAGC.

The PCR was performed for 30 cycles, followed by denaturation at 95° C for 1 min, annealing at 60° C for 1 min, and extension at 72° C for 2 min. For the second PCR, the product of the first PCR was added to the primers FV-5 GCATTCATCGACTTTCCCAC and FV-10 TAAACAGGCGGAGCACTACC.

The second PCR was performed by adding 1 µL of PCR product to the reaction mixture for 35 cycles, followed by RT reaction 50° C for 15 min, RTase inactivation at 94° C for 2 min, denaturation at 94° C for 30 sec, annealing at 46° C for 1 min, and extension at 72° C for 1 min.

**Figure 1.**
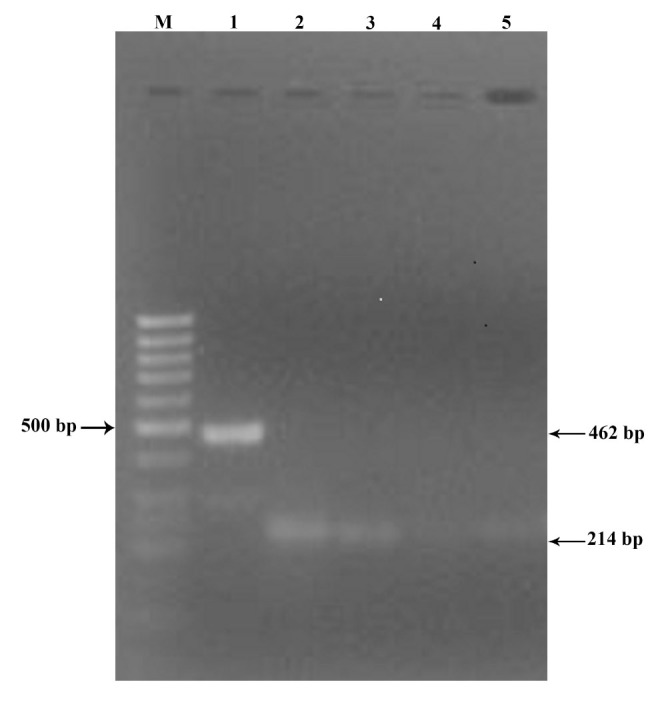
First PCR products in 1% agarose gel stained with ethidium bromide. M: Marker gene 50 bp DNA ladder; 1: Control from kit (462 bp); 2: Purified BmIFV (214 bp); 3: BmIFVinfected *Bombyx mori* midgut (214 bp); 4: DNV purified; 5: DNV-infected *B. mori* midgut tissue. High quality figures are available online.

For agarose gel electrophoresis, 10 µL of RTPCR products mixed with loading buffer were analyzed on 1% agarose gel, stained with ethidium bromide (0.5 µg/mL). A GeneRuler 50 bp DNA Ladder (Thermo Scientific, www.thermoscientificbio.com) was used as a marker containing the following 13 discrete fragments (in bp): 1031, 900, 800, 700, 600, 500, 400, 300, 250, 200, 150, 100, 50. The amplified DNA was detected using an ultraviolet transilluminator.

## Results and Discussion

**Figure 2.**
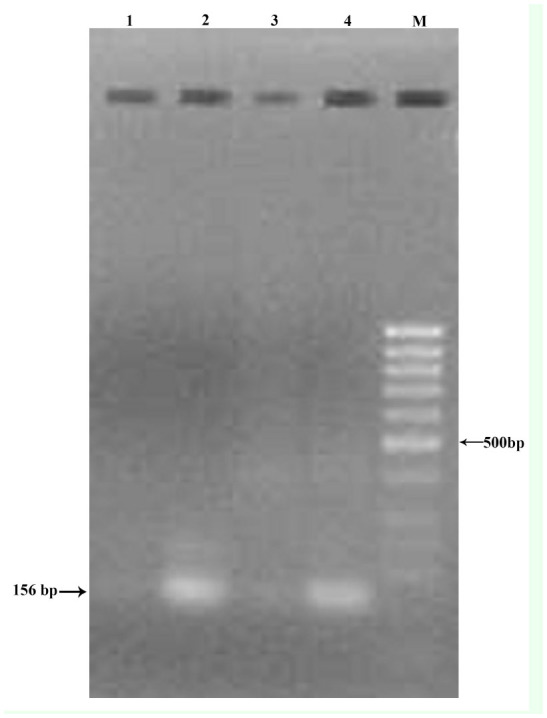
Second RT-PCR products in 1% agarose gel stained with ethidium bromide. 1: Purified DNV; 2: Purified BmIFV virus (156 bp); 3: DNV-infected midgut tissue; 4: BmIFV-infected midgut tissue extract (156 bp); M: Marker. High quality figures are available online.

**Figure 3.**
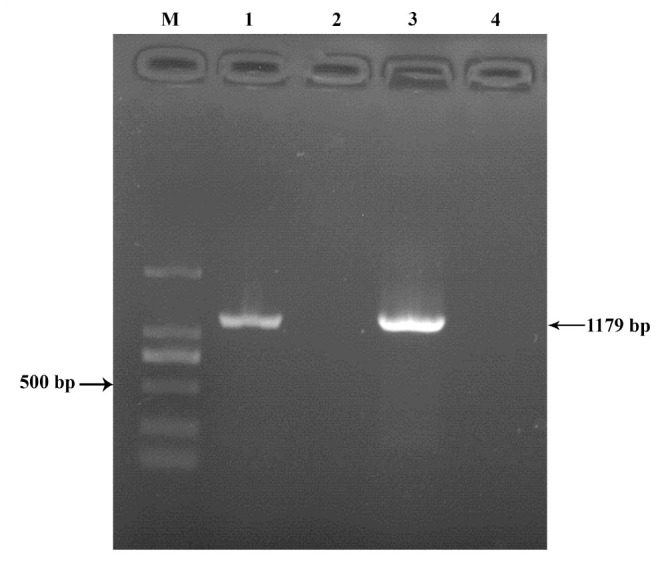
PCR amplification of DNA from DNV virus. M: Marker gene DNA ladder; 1: crude DNV(1179 bp); 2: Crude BmIFV; 3: Pure DNV (1179 bp); 4: Pure BmIFV. High quality figures are available online.

The PCR test proved positive for BmIFV using the RNA from purified BmIFV virus and BmIFV-infected tissues by using the designed primers, and the amplified PCR product of 214 bp could be visualized on Agarose gels stained with ethidium bromide (Figure 1) in both the BmIFV-infected tissue and the purified virus (lanes 2 and 3 in Figure 1), but no such observation was made with DNV virus (lanes 4 and 5 in Figure 1). In the nested RTPCR amplification of the first PCR product using the specific primer, the PCR product of 156 bp could be visualized in agarose gels (Figure 2) with only the virus (lanes 2 and 4 in Figure 2) and not with DNV virus or DNVinfected tissue (lanes 1 and 3 in Figure 2), thus confirming that the virus was indeed BmIFV. To further conclude the amplification results, negative controls were used by amplifying DNA from crude virus samples as well as purified DNV virus (Figure 3). An 1179 bp amplified product was visualized using DNVspecific primers in lanes 1 and 3 (Figure 3), while crude BmIFV virus and pure BmIFV virus did not show any amplification, thus confirming the specificity of the BmIFV detection using the specified primers for early detection.

RT-PCR technology has proven to be effective for diagnosing infectious agents (Rappolee et al. 1988; Raz et al. 1991), and the same has held true in our study focused on viral detection. Since the present technique detects the pathogen based on the specific conserved region of primer used correlating to the region in the whole sequence, similar results were obtained by using PCR for detecting BmIFV (Kageyasu et al. 1997). RTPCR has afforded many opportunities in diagnostics, allowing sensitive detection of RNA viruses such as HIV and HCV, and has benefitted molecular investigation of disease pathogenesis (O'Connell 2002). To avoid any false amplification, nested PCR was used recently for viral white spot syndrome studies (Sahul Hameed et al. 2005). Comparison of various kinds of PCR for detection of white spot syndrome has been found to be the most reliable method, and nested RT-PCR has been found to be the most fruitful (Kallaya et al. 2006). The use of technical service centers to establish a PCR and the use of high tech diagnostics for disease detection at early stages are anticipated to revolutionize the use of this technology by making the technology easily implemented at the farm level through transfer of technology programs, which are initiated by the central and state sericulture agencies by establishing PCR and testing facilities at nodal regions that are easily accessible to the farmers. This technology will hopefully provide a strategy for early detection of the diseases, which could help prevent crop loss and enable appropriate control measures to prevent spreading of pathogens.

## Abbreviations

BmIFV,: *Bombyx mori* infectious flacherie virus;
PCR,: polymerase chain reaction;
RT,: reverse transcriptase;

## References

[bibr01] Ayuzawa C, Furuta Y, Kodoma R, Nakasuji Y (1968). On the synergism between the viruses and the bacteria in the development of flacherie of the silkworm *Bombyx mori* L.. *Journal of Sericultural Science of Japan*.

[bibr02] Bongale UD (1991). Mulberry sericulture zones and agroclimate in Karnataka.. *Indian Silk*.

[bibr03] Croci L, De Medici D, Morace G, Fiore A, Scalfaro C, Beneduce F, Toti L (1999). Detection of hepatitis A virus in shellfish by nested reverse transcription-PCR.. *International Journal of Food Microbiology*.

[bibr04] Hadimani AK, Shyamala MB (1983). On the nature of ‘Kenchu” disease of the silkworm *Bombyx mori* L. in Karnataka.. *National Seminar on Silk Research & Development*..

[bibr05] Himeno M, Onodera K, Tanami Y (1974). Properties of flacherie virus of the silkworm *Bombyx mori* L.. *Journal of Invertebrate Pathology*.

[bibr06] Isawa H, Asano S, Sahara K, Iizuka T, Bando H (1998). Analysis of genetic information of an insect picorna-like virus, infectious Flacherie virus of silkworm: evidence for evolutionary relationships among insect, mammalian and plant picorna (-like) viruses.. *Archives of Virology*.

[bibr07] O'Connell J (2002). RT-PCR in Biomedicine: Opportunities Arising from the New Accessibility of mRNA.. *Methods in Molecular Biology*.

[bibr08] Kageyasu S, Hayakawa T, Isawa H, Asano S, Sahara K, Lizuka T, Bando H (1997). Detection of the silkworm pathogenic virus genomes by PCR.. *Journal of Sericultural Science of Japan*.

[bibr09] Sritunyalucksana K, Srisala J, McColl K, Nielsen L, Flegel TW (2006). Comparison of PCR testing methods for white spot syndrome virus (WSSV) infections in penaeid shrimp.. *Aquaculture*.

[bibr10] Kawase S, Hashimoto Y, Nakagaki M (1980). Characterization of infectious flacherie virus of the silkworm *Bombyx mori* L.. *Journal of Sericultural Science of Japan*.

[bibr11] Zhang L, Pan Z, Geng S, Chen X, Hu S, Liu H, Wu Y, Jiao X, Liu X (2010). Sensitive, semi-nested RT-PCR amplification of fusion gene sequences for the rapid detection and differentiation of Newcastle disease virus.. *Research in Veterinary Science*.

[bibr12] Li M, Chen X, Wu X, Man N, Jin W, Lu X (2010). Genome Analysis of the *Bombyx mori* Infectious Flacherie Virus Isolated in China.. *Agricultural Sciences in China*.

[bibr13] Dennis Lo YM (1998). *Clinical Applications of PCR: Methods in Molecular Medicine*, volume 6..

[bibr14] Mallet F, Oriol G, Mary C, Verrier B, Mandrand B (1995). Continuous RT-PCR using AMV-RT and Taq DNA polymerase: characterization and comparison to uncoupled procedures.. *Biotechniques*.

[bibr15] Nakagaki M, Takei R, Nagashima E (1987). Improved method of purification of the infectious flacherie virus and the *Bombyx* densonucleosis virus.. *Journal of Sericultural Science of Japan*.

[bibr16] Patil CS, Spence KD, Gurusiddaiah S, Kyriakides TC, Dandin SB (1992). Studies on the kenchu virus diseases of the silkworm, *Bombyx mori* L. I. purification and properties of Bng-KVI virus.. *Sericologia*.

[bibr17] Sánchez-Seco MP, Rosario D, Quiroz E, Guzmán G, Tenorio A (2001). A generic nested-RT-PCR followed by sequencing for detection and identification of members of the alphavirus genus.. *Journal of Virological Methods*.

[bibr18] Priyadharshini P, Mahalingam CA, Shashidhar KR (2008). Identification and characterization of bacterial pathogens in silkworm, *Bombyx mori* L.. *Current Biotica*.

[bibr19] Rappolee DA (1988). Wound macrophages express TGF-α and other growth factors in vivo: Analysis by mRNA phenotyping.. *Science*.

[bibr20] Raz V (1991). PCR based identification of new receptors: molecular cloning of a receptor for fibroblast growth factors.. *Oncogene*.

[bibr21] Sahul Hameed AS, Parameswaran V, Syed Musthaq S, Sudhakaran R, Balasubramanian G, Yoganandhan K (2005). A Simple PCR Procedure to Detect White Spot Syndrome Virus (WSSV) of Shrimp, *Penaeus monodon* (Fabricious).. *Aquaculture International*.

[bibr22] Samson MV, Baig M, Sharma SD, Balavenkatasubbaiah M, Sasidharan TO, Jolly MS (1990). Survey on the relative incidence of silkworm diseases in Karnataka.. *Indian journal of Sericulture*.

[bibr23] Sanakal RD, Ingalhalli SS, Singh KK, Basavarajappa S, Hinchigeri SB, Savanurmath CJ (1996). Infectious Flacherie of the silkworm *Bombyx mori* in Northern Districts of Karnataka, India.. *Indian journal of Sericulture*.

[bibr24] Savanurmath CJ, Basavarajappa S, Hinchigeri SB, Ingalhalli SS, Singh KK, Sanakal RD (1994). Relative incidence of the silkworm viral diseases in agroclimatic zones of northern Karnataka, India.. *Bulletin of Sericultural Research*.

[bibr25] Tamura K, Dudley J, Nei M, Kumar S (2007). MEGA4: Molecular Evolutionary Genetics Analysis (MEGA) software version 4.0.. *Molecular Biology and Evolution*.

